# Eddy Current Transducer with Rotating Permanent Magnets to Test Planar Conducting Plates

**DOI:** 10.3390/s19061408

**Published:** 2019-03-22

**Authors:** Tomasz Chady, Jacek M. Grochowalski

**Affiliations:** Faculty of Electrical Engineering, West Pomeranian University of Technology, ul. Sikorskiego 37, Szczecin 70-313, Poland

**Keywords:** eddy current testing, nondestructive testing, applied electrotechnics, permanent magnets

## Abstract

In this paper, we present an eddy current transducer with rotating permanent magnets for the inspection of planar conducting plates. The transducer consists of a rotating head with permanent magnets, which is used to generate variable magnetic fields and thus induce eddy currents in the tested material. Two Hall sensors connected in a differential manner are used to detect a nonuniform distribution of eddy currents induced in a specimen containing a defect. To prove the usability of the transducer, a number of experiments were conducted on thick aluminum samples containing notches at different depths. Selected results of the achieved measurements are presented.

## 1. Introduction

Eddy current testing (ECT) is a non-destructive testing technique [1,2] commonly utilized in many industries, for example, in the aerospace (e.g., detection and characterization of hidden cracks around fastener sites in multilayered structures) [3], nuclear (e.g., inspection of spent fuel canister closure welds to detect and characterize deep defects) [4], petrochemical (e.g., detection and characterization of fatigue cracks in pipelines and fuel tanks) [5] and shipyard industries (e.g., evaluation of corrosion), because of its high sensitivity, simplicity and the inexpensive instrumentation. It has been used in a range of technological applications such as surface inspection [6], quality inspection [7] or in thickness measurements [8]. 

Several types of ECT techniques have been developed so far; for example, the pulsed eddy current technique, where an eddy current probe is excited with a rectangular stimulus [9], or the single or multiple frequency technique, where the probe is excited with one or a series of sinusoidal signals [10,11]. Another approach is the Lorentz force eddy current technique (LET) [12,13,14,15]. It exploits the advantages of applying DC magnetic fields and the relative motion of the electrically conductive materials. In the LET, eddy currents are generated due to the relative motion of the conducting test object and the permanent magnet. If the magnet passes a discontinuity, the Lorentz force changes and enables the detection of defects. 

In the US Patent 2,897,438 from 1959 [16], an early application of rotating permanent magnets used in a casing joint detector was proposed. These permanent magnets generate eddy currents in casings which are used in oil wells. The joint, having a high resistance, causes a disturbance of the eddy currents and magnetic flux, which can be detected by measuring coils working in different arrangements. 

With an advancement in production technology of strong permanent magnets, it was possible to make more advanced tools for inspection of ferromagnetic objects, such as pipe and heat exchanger tubing, utilizing eddy current induced by rotating permanent magnets. In Reference [17] such system was presented. It was consisted of permanent magnets mounted on the arms (two- or four-poles magnetizer), rotating around a shaft placed inside a ferromagnetic pipe. Detection of metal loss caused by corrosion was carried out by measuring the absolute value of the magnetic field using an array of Hall sensors positioned in the specific distance from the exciter. This setup is similar to arrangement utilized in remote field eddy current transducers).

Another aspect of the eddy current NDT technique is the method of detecting disturbances of the magnetic field distribution caused by material inhomogeneities. Several types of magnetic sensors which are utilized in eddy current probes exist, such as pick-up coils [18], magnetoresistive sensors [19], Hall sensors [20], and in special cases, superconducting quantum interference devices (SQUIDs) [21]. 

The most widely used type of sensors is various kinds of pick-up coils. Different coil structures are utilized to induce eddy currents and to measure signals, namely encircling coils [22], pancake-type coils [23], horse-shaped coils (equipped with a ferromagnetic core) [24], or spiral coils with various geometries [25,26]. In many applications, arrays of probes are also utilized [27,28].

The eddy current transducers can work in an absolute mode, where only one sensing element is used, or in a differential mode, where there are two or more sensors which enable the user to compare the reaction of eddy currents in the adjacent parts of the inspected specimen [1]. The differential transducers allow for the detection of smaller-sized defects, but at the same time, long flaws or slow dimensional variations may remain undetected. Additionally, signals achieved from the differential mode transducers are more complex and their interpretation is difficult. Conversely, absolute-mode probes can detect all kinds of flaws, but they are highly sensitive to noise.

This paper presents an eddy current transducer designed to test planar conducting plates. It can also be used to inspect elements in environments where there are hazards, such as flammable gasses or combustible dust in quantities sufficient to produce fire or explosion. The introduction of high-power electrical equipment in such conditions may pose a risk because it could be a potential source of ignition; e.g., sparks caused by malfunctioning coils, stray electric and leakage currents, listed out by Directive 2014/34/Eu of the European Parliament [29]. Minimizing the use of high-power electrical devices could improve the safety of the instrument in potentially explosive atmospheres. In the case of numerous devices, the inspection is impeded due to the above-mentioned hazardous environment; for example, hydrogen generator equipment used in a hydrogen-cooled turbogenerator, gas or oil pipelines and distribution devices. In such cases, the presented transducer can be applied.

The proposed transducer utilizes a rotating head consisting of permanent magnets with radial magnetization to generate a variable magnetic field, which therefore does not require a high current excitation system. By changing the number of magnets, their arrangement and size, it is possible to achieve a specific field distribution and various frequency components. In the final design, the motor that drives the head with the magnets could either be pneumatic or hydraulic, which completely eliminates the necessity to use high-power electrical components. The two Hall sensors were used to observe the reaction of eddy currents by measuring the magnetic field intensities in an absolute and differential manner. The only electrical devices used in the proposed transducer are low-power, low-voltage, low-current Hall sensors. The concept of this transducer was presented in References [30,31] and numerical simulations of a similar simplified setup were described in Reference [32].

In contrast to the transducer shown in Reference [17], the sensor proposed in this paper allows testing flat ferromagnetic and non-ferromagnetic objects. An array of magnets mounted on the transducer head guarantees an even distribution of the magnetic field. The magnetic field sensors were placed directly under the rotating magnets, which makes it possible to detect even small defects. In the presented measuring system, the change of the rotational speed of the head, due to the Lorentz force, is primarily examined. This is achieved by measuring the frequency of the signal obtained from a single Hall sensor [30,31]. In addition, the sensing elements are connected in a differential manner to guarantee the identification of even the deepest defects. The change (reduction) of the rotational speed of the head, and thus the excitation frequency, is also used to increase the penetration depth of the magnetic field and to identify deep defects.

The fundamental working principles of the transducer are provided in Section 2. Section 3 presents the experimental setup for the testing and validating of the transducer, while Section 4 contains the experimental procedures and methodology. The results of the experiments are given in Section 5. 

## 2. Transducer and Measuring System

**Figure 1:**
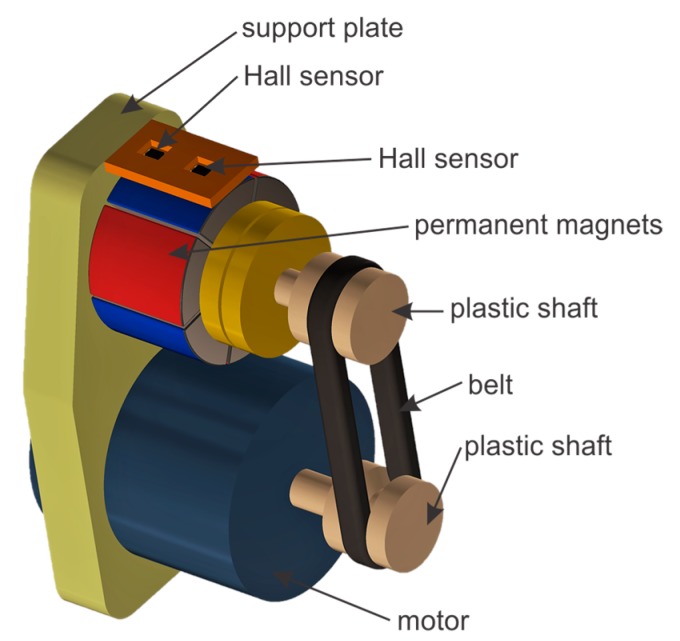
Simplified view of the eddy current transducer.

The presented transducer can be used for eddy current inspection, especially in the case of thick metal elements, because of the high field intensity and low frequency of excitation. Additionally, it can be battery operated or used in hazardous environments with the electric motor substituted with pneumatic or hydraulic rotating actuators. A simplified view of the transducer is presented in Figure 1. 

The transducer consists of the following:

- A rotating head holding permanent magnets in the form of a multipole ring with radial magnetization;
- An electric/pneumatic/hydraulic motor, which rotates the head via the plastic shaft and the belt;
- The two Hall sensors connected in a differential manner to measure the eddy current response (an absolute signal from one of the Hall sensors is also monitored simultaneously);
- A support plate which links all the elements together.

**Figure 2:**
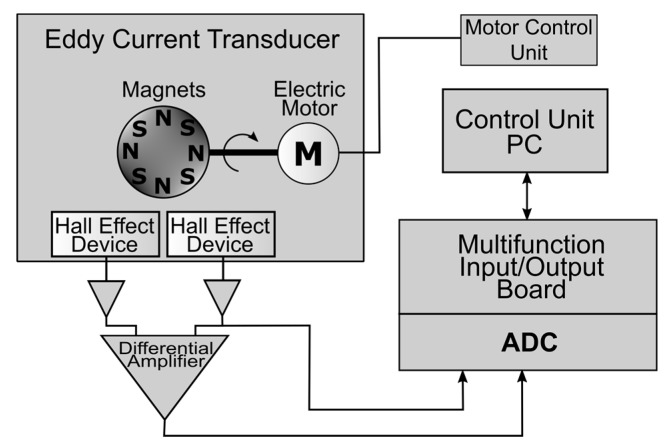
Block scheme of the measuring system.

The motor drives the revolving head with the magnets. The rotating magnets generate a variable magnetic field and subsequently induce eddy currents in the conductive test element. Field distribution over the tested element surface is monitored using the two Hall sensors connected in a differential manner. The sensors are placed between the rotating head with permanent magnets and the surface of the test sample. In the case of a test element being made of a homogeneous material, the fields acting on the two Hall sensors are nearly identical, and therefore the differential voltage will be close to zero. If a defect is present in the test specimen, an asymmetric field distribution will form and a resulting voltage difference will be generated by the respective Hall sensors. This differential signal is amplified by the instrumentational amplifier, which is then fed to the input of the analog-to-digital converter. The block scheme of the measurement system is shown in Figure 2.

**Figure 3:**
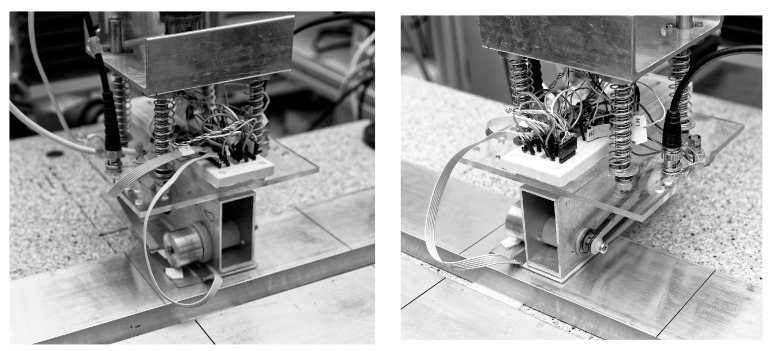
Prototype of the eddy current transducer with rotating magnets.

The rotational speed of the head is not stabilized and is affected by the reaction with the test element. Therefore, the speed is measured by one of the earlier-mentioned Hall sensors and furthermore may be used to detect the defects. All the resulting signals are converted into digital form by the data acquisition board (National Instruments PXI-5922 24-bit Digital Oscilloscope) and transferred to a desktop computer. The computer unit allows for the collection, processing and examination of the data. A cross-correlation between both signals from the Hall sensors (absolute and differential) enhances the probability of defect detection. A prototype of the proposed transducer was manufactured, and selected photos showing its construction are presented in Figure 3.

## 3. Experimental Setup and Procedure

The transducer prototype was tested to evaluate its usability. A laboratory measurement system has been set up for this purpose.

The experimental setup consists of the following:

- The transducer with rotating permanent magnets (eight poles) and an electronic interface;
- A motor control unit;
- An XY-scanner used to move the transducer over the samples;
- A desktop computer.

**Figure 4:**
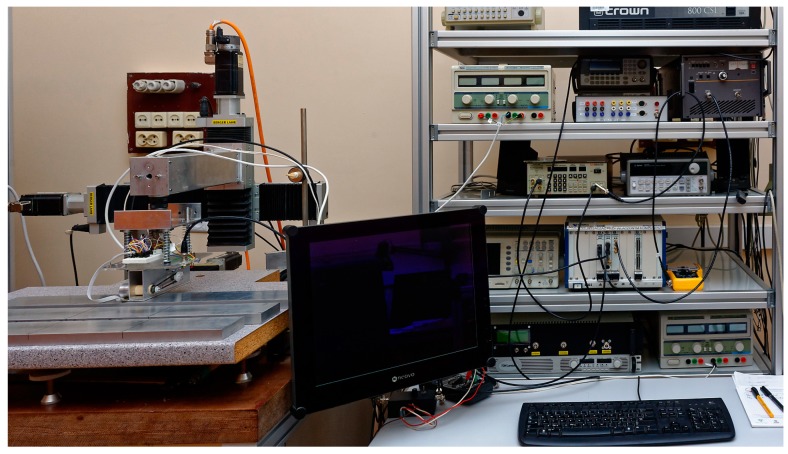
Photo of the experimental system and the aluminum test samples.

All the elements are controlled by the computer. The transducer is mechanically coupled with the XY-scanner, which moves it across the surface of the tested specimen. The program required to carry out the measurements was developed in the MATLAB environment. It commands the XY-scanner, initiates the acquisition of the signals, and processes, analyzes and presents the collected data. A photo of the experimental setup is shown in Figure 4.

The experiments were carried out using thick (20 mm) aluminum bars with various artificial defects (slits). For each specimen and defect, the experiments were executed as follows:

- The eddy current transducer head was set to move across the specimen with the Hall sensors facing the specimen surface at a distance of approximately 2 mm from the sensor;
- The defects were located in the specimen on the same side as the transducer (the inner defects);
- The transducer was slowly moved along the specimen by the XY scanner;
- There were 240 measurement points taken inline (120 measurement points on each side of the defect), with the distance between the different measuring positions being 0.5 mm;
- Both signals (differential and absolute) from the Hall sensors were acquired for each measurement point with a sampling frequency of 100 kHz, and both signals were saved for future analysis.

The acquired signals were analyzed using the software developed in the MATLAB environment. After passing the signal through a low-pass filter, the RMS (root mean square) value and the frequency deviation were computed.

The RMS value of the differential signal was calculated according to the following equation:

(1) $$\Delta U_{B,\mathrm{RMS}}=U_{B,\mathrm{RMS}0}-U_{B,\mathrm{RMS}}$$

where

- *T* is the signal period;
- *U*_B,RMS_ is the RMS (root mean square) value calculated as $U_{B,\mathrm{RMS}}=\sqrt{\frac{1}{T}\int_{0}^{T}U_{B}{}^{2}\left(t\right)dt}$;
- *U*_B_(*t*) is the Hall-effect voltage corresponding to the magnetic flux density;
- *U*_B,RMS0_ is the RMS value achieved for the position of the transducer over the homogenous material.

The frequency spectrum of the signal achieved from the single Hall sensor (after low-pass filtering) was calculated for each measurement point. Lastly, the frequency deviation was determined according to the following equation:

(2) $$\Delta f_{\mathrm{fund}.}=f_{\mathrm{fund}.,0}-f_{\mathrm{fund}.}$$

where

- *f*_fund._ is the fundamental frequency (first, lowest harmonic) resulting from the spectrum of the signal acquired at the current position of the transducer;
- *f*_fund.,0_ is the fundamental frequency resulting from the spectrum of the signal acquired at the position of the transducer above the homogenous material.

As mentioned earlier, the rotational speed of the motor and the head is not stabilized. Therefore, when the specimen under the transducer contains a defect, the Lorentz force changes and the rotational speed of the head is affected. A variation of the head’s rotational speed can be detected by observing the fundamental frequency of the signal coming from the Hall sensor.

## 4. Results of Experiments

**Figure 5:**
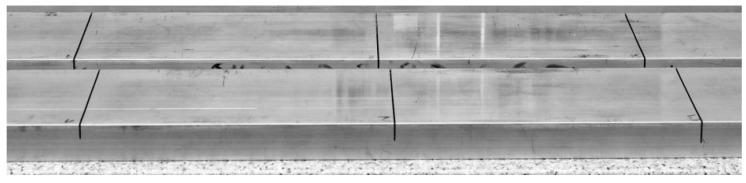
Photo of the aluminum samples with manufactured notches.

The experiments were conducted on a set of aluminum flat bars. Each specimen was 80 mm wide and 20 mm thick. On each of them, three defects (notches) were manufactured, each with a width of 0.7 mm, using a precisely controlled saw. Table 1 contains a detailed description of all tested samples, and one of the samples is shown in Figure 5.

### 4.1. Comparison of the Results Achieved at Different Defect Depths

**Figure 6:**
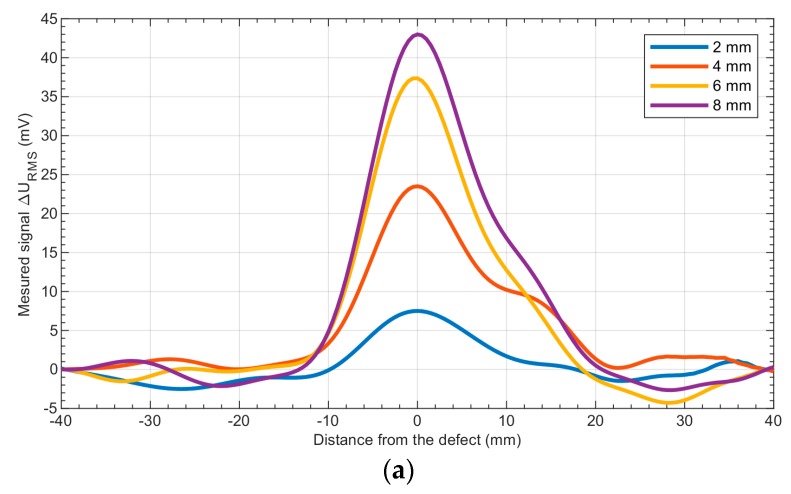

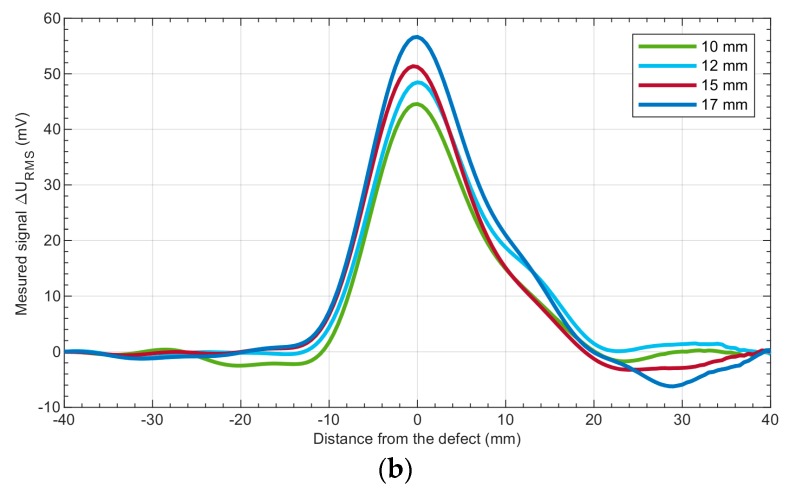
Results of the measurements obtained for aluminum samples—a comparison of the signals (Root Mean Square values—RMS) for different depths of the notches: (**a**)—2–8 mm, (**b**)—10–17 mm. The angular speed of the head was 3700 rpm.

The RMS value of the acquired differential signal ∆*U*_B,RMS_ was calculated for all the defects with the transducer head at three different angular speeds (2600, 3700, and 4800 rpm). Figure 6 shows a comparison of the achieved RMS signals for various defect depths, measured at the same rotational speed.

**Figure 7:**
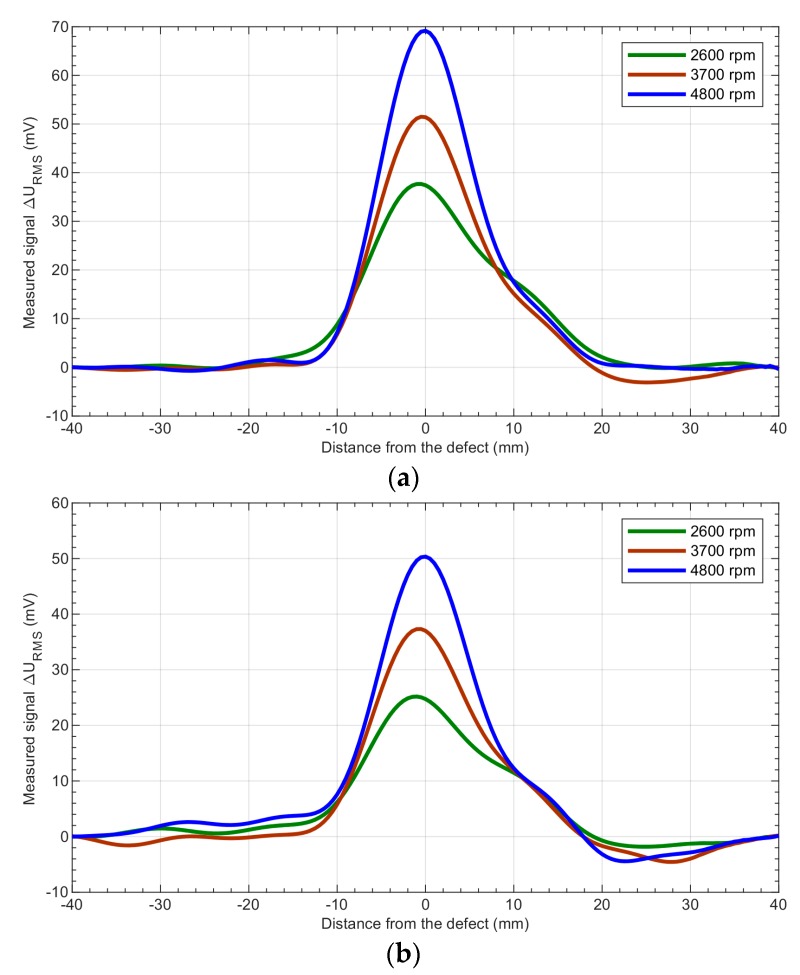

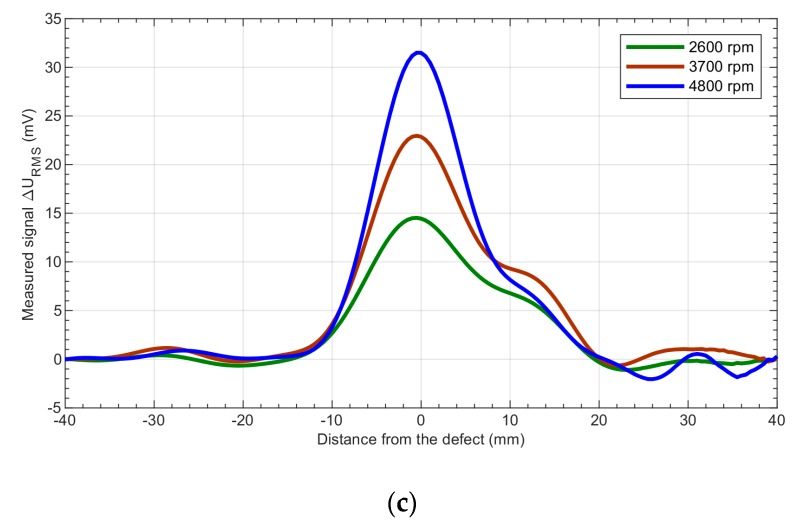
Results of the measurements executed for the aluminum samples with notches having depths of (**a**) 15 mm, (**b**) 6 mm, (**c**) 4 mm—comparison of the measured signals achieved at the different rotational speeds: 2600 rpm, 3700 rpm, and 4800 rpm.

As is shown in Figure 6, the position and depth of the defects can be estimated from the results. It should be noted that the response achieved is not symmetrical and the signal begins to rise 10 mm before the defect and then decreases to the previous value 20 mm after the defect. The asymmetry of the signal is caused by the nonsymmetrical distribution of the eddy currents induced in the specimen by the head rotating in one direction only. Figure 7 shows the relationship between the rotational speed of the head and the depth of the defects obtained at different angular speeds of the transducer. 

**Figure 8:**
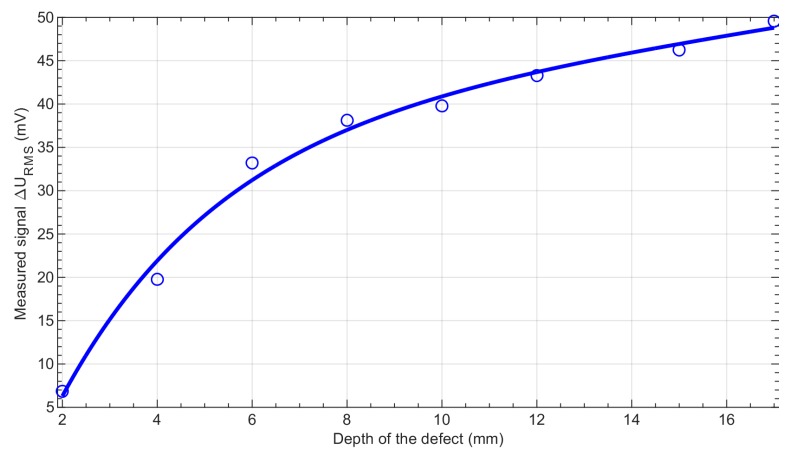
Maximum values of the signals measured for the aluminum samples with defects having different depths (solid line—approximation curve, circles—exact measured values).

Figure 8 displays the interpolated relationship between the measured maximum value of ∆*U*_B,RMS_ and the depth of the notch. The calibration curve allows for the opportunity to estimate the depth of the detected defect.

### 4.2. Observation of the Fundamental Frequency Deviation Achieved for Different Defect Depths

**Figure 9:**
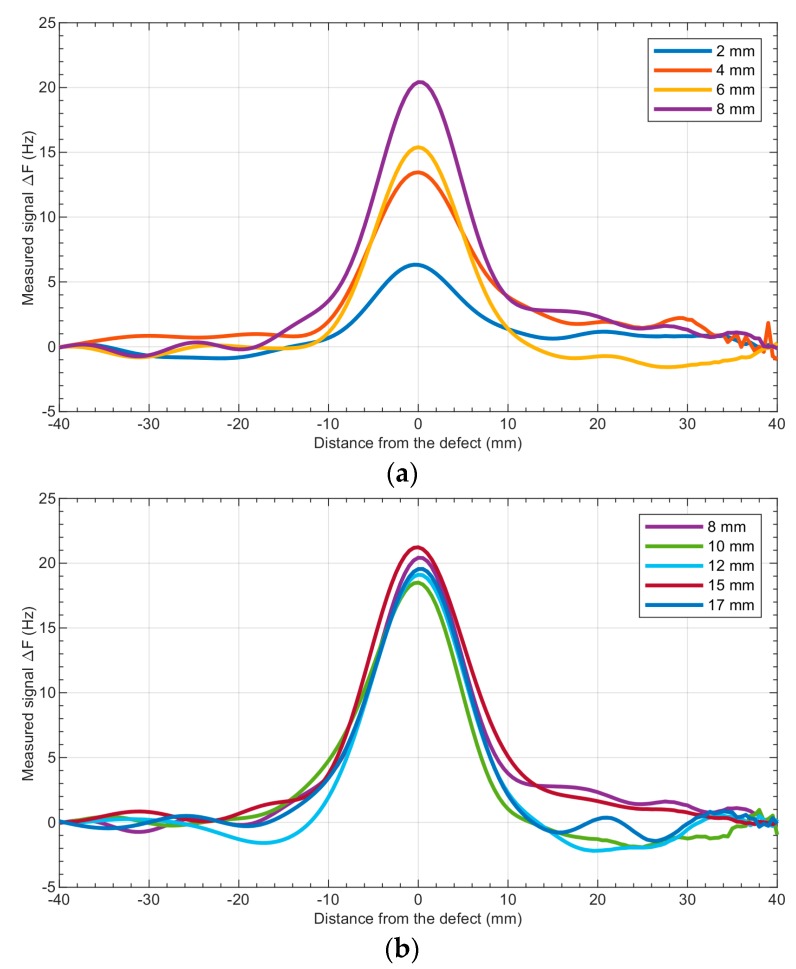
Results of the measurements for the aluminum sample—comparison of the measured signal (the deviation of the fundamental frequency ∆*f*_fund._) for different depths of defects: (**a**)—2–8 mm, (**b**)—8–17 mm.

The next series of experiments were carried out using frequency spectrum analysis in order to analyze the deviation of the fundamental frequency. The samples previously used were also utilized in this experiment. Figure 9 shows a comparison of fundamental frequency deviation for various defect depths. The position of the defect is clearly noticeable. The increased signal value is observed within 10 mm before and after the defect, and the resolution is, therefore, better than in the case of the RMS value observation. Moreover, the measured signal is central with respect to the defect position.

**Figure 10:**
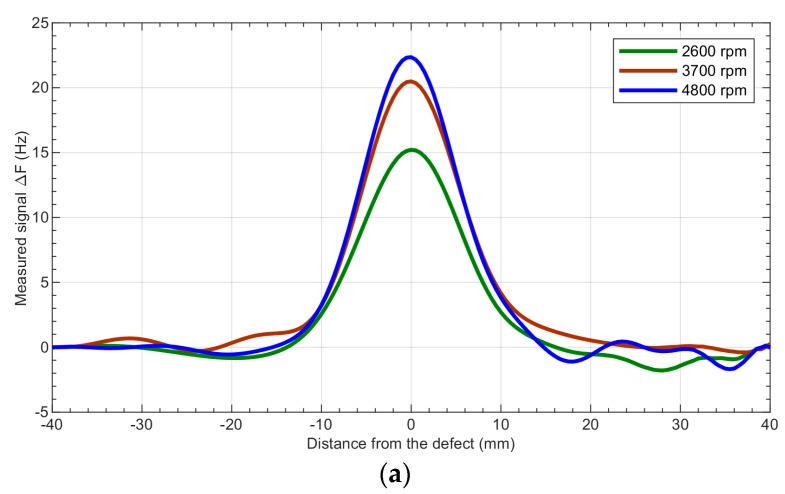

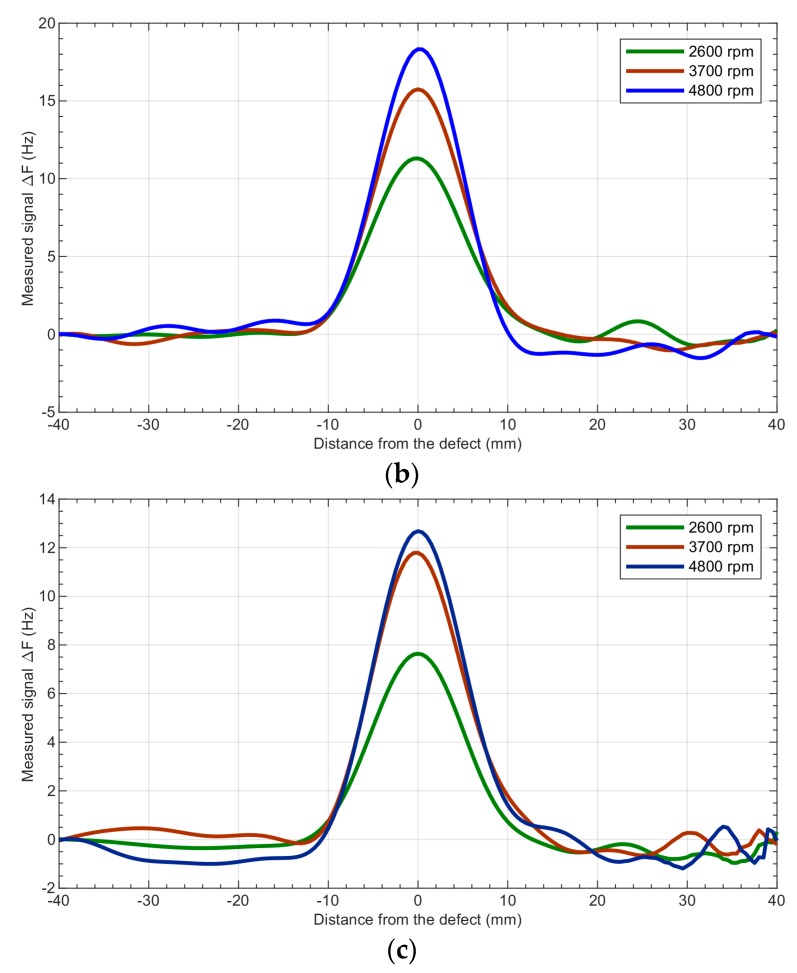
Results of the measurements acquired from the aluminum sample with notches having depths of (**a**) 15 mm, (**b**) 6 mm, and (**c**) 4 mm—comparison of the deviation of the fundamental frequency for different rotational speeds of the head: 2600 rpm, 3700 rpm, and 4800 rpm.

Figure 10 shows the influence of the head’s rotational speed on the fundamental frequency deviation caused by different defects.

**Figure 11:**
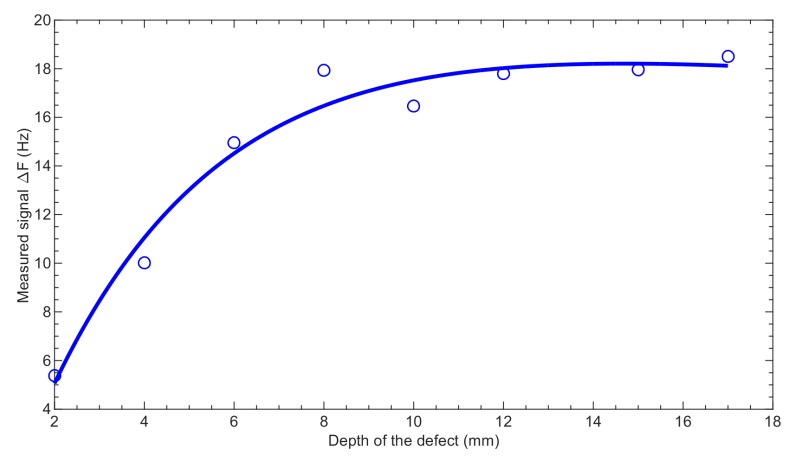
Maximum values of the deviation of the fundamental frequency measured for the aluminum samples with defects having different depths (solid line—approximation curve, circles—exact measured values).

Figure 11 shows the interpolated relationship between the maximum measured ∆*f*_fund._ value and the depths of the defects. A saturation effect can be observed for the larger notch depths. In the case of the differential voltage from the Hall sensors (∆*U*_B_), the flaw depth can be clearly distinguished up to 17 mm, while in the case of the fundamental frequency, this is possible only up to 10 mm (Table 2).

## 5. Conclusions

The flat aluminum specimens with defects were inspected using the eddy current transducer with rotating permanent magnets as presented in this article. A dedicated experimental setup was built to test the transducer, the hardware (XY—scanner, signal acquisition system) and the software, using the MATLAB environment. 

Different sets of samples with varying defects—notches of various depths—were tested. All the defects were shown to be clearly detected using the differential signal achieved from the two Hall sensors as well as the deviation of the fundamental frequency. The depth of the defects could also be identified from the measured signals. The RMS value of the differential signal is particularly useful in estimating the depth of the defect. In this instance, even defects with the highest depths can be clearly identified (the calibration curve does not tend to saturate). The deviation of the fundamental frequency is more useful for position estimation because the response of the defect is nearly symmetrical and its width narrower.

The proposed eddy current transducer lacks excitation coils and can operate in hazardous environments in combination with a hydraulic or pneumatic motor; for example, in environments where substances are in large enough quantities to start a fire or trigger an explosion. It can be used to test planar conducting surfaces such as the walls of metal containers or pipes and may be battery operated, making it a versatile eddy current inspection tool. Additionally, using a similar principle, it is possible to construct an eddy current transducer without any electrical circuits [33]. 

It is to be hoped that, the presented here solution with differential detection elements and self-adaptive mechanism for generating variable frequency eddy currents will contribute to the further development of eddy current transducers with rotating permanent magnets, especially in case of sensors designated for testing of non-ferromagnetic and flat objects.

## Figures and Tables

**Table 1:** Comparison of the tested samples.

	Sample A	Sample B	Sample C
Length of the sample [mm]	650	650	690
Defect depth [mm]	2, 4, 6	8, 10, 12	15, 16, 17

**Table 2:** Comparison of relative changes in the signal value for various defects in relation to the defect with a depth of 17 mm.

Flaw depth [mm]	2	4	6	8	10	12	15
∆*U*_B_*/*∆*U*_B_^(17mm)^ [%]	90.5	57.3	37.5	25.1	16.9	10.9	4.0
∆*f*_fund/_∆*f*_fund_^(17mm)^ [%]	72.6	39.8	20.7	9.7	4.2	1.5	0.5
